# Psychological Support Interventions for Healthcare Providers and Informal Caregivers during the COVID-19 Pandemic: A Systematic Review of the Literature

**DOI:** 10.3390/ijerph18136939

**Published:** 2021-06-28

**Authors:** Vanessa Bertuzzi, Michelle Semonella, Denise Bruno, Chiara Manna, Julian Edbrook-Childs, Emanuele M. Giusti, Gianluca Castelnuovo, Giada Pietrabissa

**Affiliations:** 1 Department of Psychology, Catholic University of Milan, 20123 Milan, Italy; vanessa.bertuzzi@unicatt.it (V.B.); denise.bruno01@icatt.it (D.B.); chiara.manna02@icatt.it (C.M.); gianluca.castelnuovo@unicatt.it (G.C.); 2 Department of Psychology, Bar-Ilan University, Ramat-Gan 52900, Israel; michelle.semonella@biu.ac.il; 3 Evidence-Based Practices Unit, University College London and Anna Freud National Centre for Children and Families, London N1 6EB, UK; Julian.Childs@annafreud.org; 4 Psychology Research Laboratory, Istituto Auxologico Italiano IRCCS, 20145 Milan, Italy

**Keywords:** COVID-19, healthcare providers, informal caregiver, psychological support intervention, digital psychological intervention, clinical psychology

## Abstract

Background: During the COVID-19 pandemic, healthcare providers and informal caregivers were at an increased risk of adverse mental health effects. This systematic review provides a summary of the available evidence on the content and efficacy of the psychological support interventions in increasing mental health among healthcare providers and informal caregivers during the COVID-19 pandemic. Methods: PubMed, Google Scholar, PsychINFO, and Scopus databases were systematically searched for relevant articles, and the methodological quality of selected articles was assessed using the Quality Assessment Tool for Quantitative Studies. Results: A search of electronic databases identified five reports based on inclusion and exclusion criteria. All psychological support interventions for caregivers were delivered digitally. Despite the large heterogeneity of the selected studies, the findings support the efficacy of mental health interventions in reducing distress and burnout, while promoting self-efficacy and well-being in both healthcare providers and informal caregivers. Conclusion: Since mental health problems are expected to increase during, and as a result of, the COVID-19 pandemic, and digital tools might offer a range of mental health treatments to meet the unique and immediate needs of people, further research is needed to test the cost-effectiveness of digital psychological interventions.

## 1. Introduction

Coronavirus disease 2019 (COVID-19) has been recognized as a Public Health Emergency of International Concern by the World Health Organization [1]. Restrictive measures, such as social distancing, the use of face masks, and mobility restrictions, have been adopted by governments worldwide to reduce the spread of the virus [2], drastically altering people’s lives [3,4]. Indeed, the uncertainties and fears associated with the virus outbreak, and the lack of clear information, together with social isolation and the consequent economic recession [5], has had a strong impact on both individuals and society as a whole (i.e., the economy and healthcare system) [2,6,7,8]. Since the onset of the pandemic, studies have reported negative health outcomes related to social isolation, including the uptake of maladaptive behaviors (i.e., an unhealthy diet and sedentary lifestyle, and the consumption of drugs and alcohol) [9] and increased levels of anxiety, worry, irritability, depressive symptoms, and post-traumatic stress disorder across a range of populations [10,11].

Caregivers may have been particularly vulnerable to the consequences of the pandemic, even if not necessarily to the effects of the virus itself [12]. A caregiver is defined as being the person that is responsible for assisting a sick or dependent person from performing the practical tasks and activities of daily living [13]. With 34% of caregivers commonly reporting depressive symptomatology and 43.6% reporting high levels of anxiety, providing care has already been proven to be a stressful experience that may negatively impact the psychological well-being of carers [14].

In unprecedented times, such as the COVID-19 crisis, the task of caring, which is generally uninterrupted, became extremely challenging for caregivers as a result of the loneliness brought about by the confinement and isolation imposed by governments [15]. In addition, the overload of activities led to an increase in stress and burden among caregivers [16]. Studies revealed that, during the COVID-19 pandemic, care providers exhibited a decrease in physical and mental health [12], and that social isolation heavily affected multiple aspects of the quality of life of caregivers [17,18], including an increase in depression and anxiety [19], besides compromising their ability to take care of others [20].

Caregivers can be both formal and informal. Formal caregivers are healthcare professionals (HCPs) who are paid for the care and support they provide to patients or clients [21]. Informal caregivers (ICs) are relatives, friends, or neighbors who provide unpaid practical support, generally in the home environment, for an aging parent, spouse, other relative, or unrelated person, or for an ill or disabled person [22], and are an essential, but often overlooked, component of the healthcare system.

HCPs, due to prolonged work shifts, isolation, fear of being infected or of infecting others, and distress over the loss of many patients and colleagues [23,24], experienced unparalleled COVID-19-related stress, emotional pain, insomnia, exhaustion, job dissatisfaction [25,26,27], and depressive symptoms [28], which are expected to increase up to several years after the outbreak [29,30].

Moreover, quarantine measures enacted to slow the viral spread resulted in limited educational (i.e., schools) and healthcare services (i.e., hospitals, outpatient clinics) for people in need of care. ICs were required to step in to fill in the care gaps, thus replacing discontinued formal care with informal care [31], which had a negative impact on their mental well-being [32].

Caring for a significant other can be a rewarding experience, but due to a lack of time and energy, or financial, emotional, and social strains, it can also turn out to be an overwhelming responsibility for caregivers.

In this scenario, independent of whether the care is formal or informal, it is important to evaluate and minimize the burden of care providers, and to support them with tailored and integrated healthcare actions. In fact, despite the fact that care providers differ in their working style, motivations, and aims between formal and informal caregivers, COVID-19 brought about overlapping responsibilities and a shift in roles that led both populations of caregivers to experience increased and imbalanced care distribution that negatively affected their mental well-being [33,34]. They were not only exposed to higher levels of stress, anxiety, and depression [35,36], but also equally revealed sleep disturbances [37], frustration, and hopelessness during their caregiving roles, with the consequent risk of struggling with negative emotions, such as self-criticism and shame [38,39].

To this aim, psychological interventions have been developed to support HCPs [24,32] and informal caregivers [3,40,41] during the COVID-19 pandemic. However, there is a lack of systematic evaluations of existing mental health management strategies for caregivers from the current pandemic, thus limiting the conceptualization of timely and effective actions of care.

To overcome this gap, the present systematic review provides a summary of the evidence for the available psychological support interventions and strategies, and their impact on psychological health, for both HCPs and informal caregivers, during the first and second waves of the COVID-19 pandemic.

## 2. Materials and Methods

The protocol of this systematic review was registered with PROSPERO (ID: CRD42021237827). Data extraction, critical appraisal, and qualitative synthesis were in line with established systematic review and qualitative synthesis methods [42]. This report follows the Preferred Reporting Items for Systematic Reviews and Meta-Analyses (PRISMA) statement [43].

### 2.1. Search Strategy

Searches were conducted in the following databases: PubMed, Scopus, PsychINFO, and Google Scholar, between 26 February and 28 February 2021. The two search strategies (one for HCPs and one for ICs) combined key terms and Medical Search Headings (MESH) terms based on the patient problem (or population), intervention, comparison (or control), and outcome in accordance with the PICO [44] elements, as follows:

- (“informal caregiver” OR parent*) AND (“emotional support” OR “psychological support” OR “psychological assistance”) AND (Covid-19) AND (“well-being” OR “mental health” OR “quality of life” OR anxiety OR depression OR distress OR psych*).
- (“health care provider” OR “formal caregiver”) AND (“emotional support” OR “psychological support” OR “psychological assistance”) AND (Covid-19) AND (“well-being” OR “mental health” OR “quality of life” OR anxiety OR depression OR distress OR psych*).

### 2.2. Inclusion and Exclusion Criteria

Articles were included if they: (1) were original research articles, (2) employed quantitative methods, (3) tested the impact of a psychological intervention developed during the COVID-19 pandemic, (4) reported at least one psychological primary outcome, (5) focused on HCPs and/or informal caregivers of adults with/without mental health problems (i.e., elderly people), (5) focused on the parenting or caregiving of children and/or adolescents with mental disorders, (6) delivered education, support, or monitoring interventions.

Studies were excluded if they: (1) reported only biomedical data, (2) focused on the parenting or caregiving of children and/or adolescents without mental disorders, (3) were not original studies (i.e., epidemiological studies, opinion or prospective studies, theoretical case studies, protocol studies). No limitations were set for gender, sample size, the type of quantitative study, language, ethnicity, or the age of the care recipient.

The reference lists of all selected articles and retrieved systematic reviews were manually screened to identify any further contribution for possible inclusion, but none were found.

### 2.3. Study Selection

Following the search and exclusion of duplicates, two reviewers (authors V.B. and D.B.) independently screened the eligibility of the articles, first on the Title and the Abstract, and then the full text, according to the inclusion criteria. Disagreements were resolved by another reviewer (M.S.). Following Smith et al. (2011) [45], the review team included at least one person with methodological expertise in conducting systematic reviews (G.P. and J.E.-C.) and at least two experts on the topic under review (G.C. and G.P.). Searches of electronic databases identified 88,621 reports. Of these, 25 were duplicates and 88,589 records were excluded based on information from the Title and Abstract for the following main reasons: (1) they were not carried out during the COVID-19 pandemic, (2) they focused on the parenting or caregiving of children that did not suffer from a mental health problem, (3) they were not original articles, but case studies, opinion, or perspective studies, (4) they reported only biomedical outcomes.

The remaining seven records were evaluated for inclusion by reviewing their full texts and resulted in the exclusion of two articles for the following reasons: the psychological intervention was not provided during the COVID-19 pandemic/focused on COVID-19-related psychological problems (n = 1) [46] and did not provide psychological outcomes (n = 1) [47].

**Figure 1:**
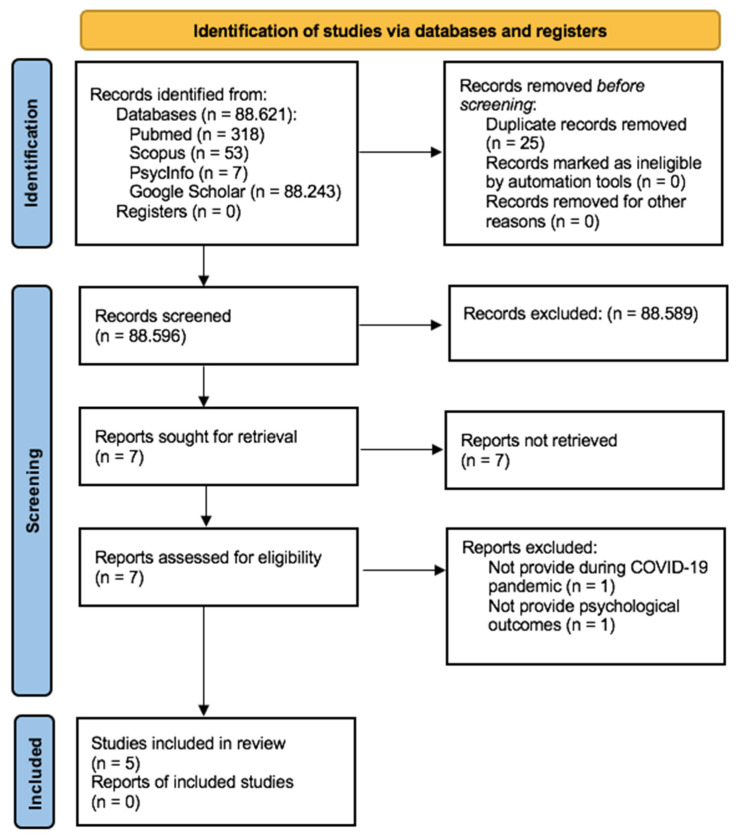
PRISMA flowchart.

Five records ultimately entered the systematic review [3,24,32,40,41]. The flowchart presented in Figure 1 provides step-by-step details of the study selection process.

### 2.4. Assessment of Risk of Bias

The Quality Assessment Tool for Quantitative Studies Dictionary [48] was used to assess the methodological quality of each selected study. This standardized tool was developed in order to provide high-quality systematic reviews to address the public health sector’s need for evidence to support practice. The final results of the quality assessment led to an overall methodological rating of strong, moderate, or weak in six sections: (1) selection bias, (2) study design, (3) confounders, (4) blinding, (5) data collection method, and (6) withdrawals/dropouts. Guidelines for the tool indicate that each section be rated as strong (3 points), moderate (2 points), or weak (1 point), and domain scores are averaged to provide the total score. The maximum total score per study is 3.00. Based on their total score, studies are assigned a quality rating of weak (1.00–1.50), moderate (1.51–2.50), or strong (2.51–3.00).

The assessment was conducted independently by two reviewers (D.B. and C.M.) and any disagreements were resolved by a third reviewer (author V.B.).

**Figure 2:**
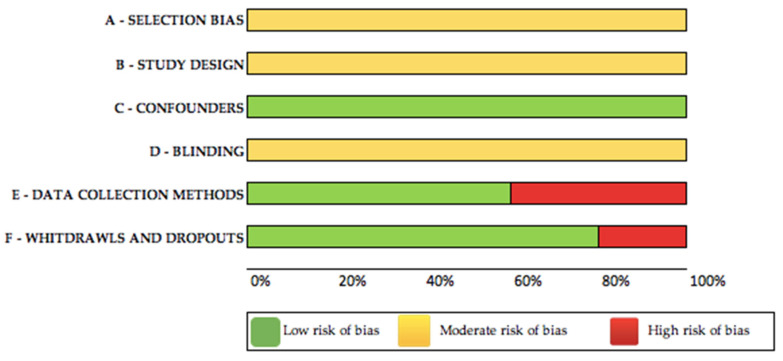
Bias Analysis Graph.

Three out of the five selected articles [3,24,32] had a moderate methodological quality (one weak rating), while the remaining two studies [40,41] showed a strong methodological quality (no weak ratings). The rating of each selected study is presented in Supplementary Material Table S1, while a Bias Analysis Graph is presented in Figure 2.

### 2.5. Data Extraction and Synthesis

Authors V.B. and D.B. independently extracted the following data from the included studies: (1) the first author and year of publication, (2) country, (3) study aim and design, (4) type and format of the delivery of psychological interventions, 5) recipients, (6) sample size, gender, and age, (7) follow-up points, (8) psychological outcomes and measures, and (9) main results. The characteristics of the included studies are reported in Table 1. Disagreements were resolved by a third author (author M.S.).

The extracted data were collated to produce a narrative summary of study characteristics to address the review question.

Considering the heterogeneity of the tools and interventions proposed by the studies, it has not been possible to perform a meta-analysis.

## 3. Results

### 3.1. Characteristics of the Included Studies

The selected studies were conducted in China [3,24,40] and Italy [32,41]. Three contributions made use of a one-group, pre–post study design [24,32,41], while in two records [3,40] a pre–post comparative design was adopted. The sample size varied widely among studies, from a minimum of 34 [32] to a maximum of 508 [3] participants. All the investigations included participants of both genders, with a mean age ranging from 31 [32] to 72 years [40]. The length of the intervention ranged from 4 [3] to 12 weeks [41]. The recipients of support interventions were HCPs assisting COVID-19 patients in two studies [24,32] and informal caregivers assisting people suffering from mental disorders in three studies [3,40,41].

### 3.2. Psychological Support Interventions for HCPs

Two studies applied a one-group, pre–post design to evaluate the efficacy of psychological support interventions in promoting well-being among HCPs [24,32]. The interventions were in digital format, but the study of Cheng et al. (2020) also included face-to-face interactions. This comprised five cognitive modules of daily measurement of mood, self-feedback training, peer-group psychological support and education, weekly Balint groups, and after-work tailored activities [24]. All HCPs maintained an overall positive outlook for nearly 6 weeks of continuous working, and significant treatment effects on gains (*p* < 0.001) and a reduction in the reported problems were also observed [24].

Giordano et al. (2020) applied a receptive music therapy treatment based on listening to two different playlists (a Breathing Playlist to favor relaxation and reduce anxiety and stress, and an Energy Playlist to recover energy and support concentration) [32]. Both the Breathing Playlist and the Energy Playlist produced lower perceived levels of tiredness, sadness, fear, and worry intensity between the baseline and the 1-week follow-up (*p* < 0.05), and a further improvement was also observed between the 1-week and 5-week follow-up with similar trajectories between the playlists.

### 3.3. Psychological Support Interventions for Informal Caregivers

All psychological support interventions for informal caregivers were delivered in digital format, but the type of treatment, digital service, and care recipients varied across studies: De Luca et al. (2020) proposed a cognitive and sensory–motor intervention to lower distress in a group of caregivers of adults with severe acquired brain injury, Guo et al. (2020) compared the efficacy of an educational program in reducing depression and anxiety among the carers of youth with or without an eating disorder, while Lai et al. (2020) compared the efficacy of different telehealth services (video-conferencing platforms vs. phone calls) in increasing the quality of life and self-efficacy of caregivers of adults with a neurocognitive disorder.

Two studies [40,41] measured the perceived burden of the caregivers using the Zarit Burden Interview Scale (ZBI) and revealed significantly increased outcomes (*p* < 0.0001) at 4-week [40] and 12-week follow-ups [41], respectively. Additionally, the ZBI anxiety scores decreased significantly (*p* < 0.0001) in one study [41], while no significant reduction in anxiety and depressive levels were registered from the baseline to the 4-week follow-up using the Generalized Anxiety Disorder-7 and the Patient Health Questionnaire-9 in the study by Guo et al. (2020). Still, the analysis revealed that, irrespective of condition, caring for patients with a shorter illness duration facilitated the reduction in symptoms of anxiety (*p* = 0.041) and that being a caregiver of an older patient (*p* = 0.037), or not living with the care recipient (*p* = 0.006), enabled a greater reduction in depressive levels [3].

Moreover, Lai et al. (2020) showed greater health-related, quality-of-life, and self-efficacy improvements among participants receiving the telehealth intervention using the video-conferencing platform compared with those receiving psychological support via phone calls after one month from the termination of treatment [40].

## 4. Discussion

To our knowledge, this is the first study aimed at providing a summary of the available evidence on the efficacy of psychological interventions to support HCPs and informal caregivers during the first and second waves of the COVID-19 pandemic.

Interestingly, despite research conducted during previous outbreaks, including SARS and the A/H1N1 virus (swine flu), revealing negative short- and long-term outcomes on the physical and mental health of individuals that are similar to those observed during the current pandemic [49,50,51,52], no pandemic-specific intervention in support for HCPs and the mental health of informal caregivers has been developed, but only guideline and preventive indications [51,53]. This might be due to the fact that social isolation prevented one from drawing on and delivering psychological support intervention. Instead, findings from this review reveal that different psychological support interventions have been developed during the COVID-19 pandemic for both HCPs and informal caregivers, and that the digital format was a feasible therapeutic solution to improve individuals’ well-being while preventing the risk of contagion and increasing compliance to treatment. This is probably due to technological progress and the increase in digital solutions within the therapeutic context. In fact, among the five studies included within this systematic review, only one was based on a mixed program delivered both digitally and face to face [24]. The use of digital solutions to improve the mental health of HCPs and ICs has been proven to be a useful format in emergency settings, such as the one characterizing the COVID-19 pandemic.

Psychological support interventions developed for HCPs were aimed at promoting positive emotions, maintaining teamwork efficacy, preventing burnout [24], reducing distress, and improving well-being [32]. Interventions developed for ICs were focused on reducing distress [41], depression, and anxiety [3], while improving well-being [41], quality of life, and self-efficacy [40]. The results revealed that digital psychological interventions were not only feasible, but also efficient in providing psychological support.

Consequently, it is evident that emotional suffering might be either similar or different between the two populations of caregivers, but also that several contemporary psychological approaches were effective in offering prompt support to carers.

### 4.1. Strengths and Limitations

Although a very extensive review covering established health and psychological databases has been performed (grey literature searches have not been performed), only five studies fully met the inclusion criteria of the present review, three of which had a moderate methodological quality and none of the contributions comprised a follow-up longer than 3 months, thus preventing the drawing of valid conclusions over the short- and long-term impact of the psychological treatments.

Moreover, the selected studies were only conducted in China and Italy, which notably were the first affected countries worldwide. This is interesting considering that they were the first affected countries worldwide but limited the generalizability of the research findings. The outcome measures also differed consistently across studies and populations, further preventing the drawing of valid conclusions on which interventions might better work for specific mental health issues.

In addition, the interventions varied widely in terms of the treatment delivered, length of the therapy, and digital tools among the selected studies. This heterogeneity precludes speculation on the superiority of a specific treatment or digital tool.

Nevertheless, the findings of this systematic review support the feasibility and adaptability of digital tools in providing psychological interventions and suggest a range of strategies that could be used to increase the emotional health of both formal and informal caregivers.

### 4.2. Future Research and Practical Directions

Studies have already demonstrated the advantages of the use of digital formats within the psychological context, including decreasing wait-lists and time, increasing cost-effectiveness [54,55], reducing geographical barriers between the patient and the therapist [56], and facilitating the disclosure of people while in their environment (i.e., their home or office) [57].

However, a few aspects should be carefully considered while developing and evaluating the efficacy of digital psychological interventions. First, it is important to conduct a usability test to evaluate whether people find the intervention platform easy to use and functional, as usability problems or ineffective systems might affect the magnitude of the treatment effect [58,59]. Additionally, the digital literacy of participants should be properly evaluated and addressed before starting the intervention. None of the selected contributions seemed to have conducted a system usability test before the delivery of the intervention, nor were the individuals tested on their ability to use digital platforms. The evidence exists for the feasibility, acceptability, and cost-efficacy of digital mental health interventions in youth in non-crisis times [60]. Still, older people might experience difficulties in the use of digital tools, and social isolation can pose additional challenges (i.e., chaotic home environments, limited privacy, or unreliable internet connection). Further studies should carefully evaluate and review the outcomes of the psychological digital interventions for formal and informal caregivers to allow for shared decision making in treatment planning to prevent or reduce the mental health burden experienced by caregivers. Digital psychological interventions for caregivers are effective, but some interventions have primarily domain-specific effects rather than global effects on both formal and informal caregivers. The differences between the intervention types suggest ways of optimizing care.

## 5. Conclusions

Due to the COVID-19 pandemic and its related social isolation, negative health outcomes have been reported across a range of populations. Particularly, HCPs and ICs have been largely affected by the ongoing pandemic. Findings from this study shed light on the evidence-based and practical solutions that these two target populations of care providers might use in order to find a release from the unique challenges that they had to face amid COVID-19. Furthermore, the results from this study may offer a valuable insight for future research and the development of psychological support interventions using digital solutions to cope with the emotional needs of caregivers.

## Figures and Tables

**Table 1:** Legend (in alphabetical order): BP = Breathing Playlist, CG = Control Group, DMI = Daily Mood Index, ED = eating disorder, EP = Energy Playlist GAD-7 = Generalized Anxiety Disorder-7, GIS = Gain-and-Issue Scale, HCP = healthcare provider, IG = Intervention Group, MD = mental disorder, MTC = Music Team Care, NCD = neurocognitive disorder, PHQ-9 = Patient Health Questionnaire-9, PSS = Perceived Stress Scale, QoL = quality of life, RCSES = Revised Caregiving Self-Efficacy Scale, SARI = severe acquired brain injury, SAS = Zung Self-Rating Anxiety Scale, SF-36v2 = Short Form 36 version 2, SSRS = Social Support Rating Scale, SUF = Subjective Units of Feeling Scale, ZBI = Zarit Burden Interview Scale.

Author, Year	Country	Study Design	Study Aim		Format	Recipients	Type of Intervention
**HEALTHCARE PROVIDERS (HCPs)**							
Cheng et al., 2020	China	One group pre–post study	To evaluate the efficacy of a psychological support intervention in promoting positive emotion, maintaining teamwork efficiency, and preventing burnout		Digital (online chat) and in person	HCPs	Five cognitive modules
Giordano et al., 2020	Italy	One group pre–post study	To evaluate the efficacy of a psychological support intervention in reducing distress and improving well-being		Digital (playlist on a mobile phone)	HCPs	Receptive music therapy
INFORMAL CAREGIVERS							
De Luca et al., 2021	Italy	One group pre–post study	To evaluate the efficacy of a psychological support intervention in reducing distress		Digital (online Skype therapy)	Informal caregivers of adults with a SARI	Cognitive and sensory–motor intervention
Guo et al., 2020	China	Pre–post comparative study	To compare the efficacy of a psychological support intervention in reducing depression and anxiety in caregivers of youth with/without eating disorders		Digital (online using WeChat or e-mail)	Informal caregivers of youths with EDs (IG) vs. informal caregivers of youths without MDs (CG)	Educational program on caregivers’ skills
Lai et al., 2020	China	Pre–post comparative study	To compare the efficacy of digital psychological support interventions to increase quality of life and self-efficacy		Digital (video conferencing platform (IG), phone calls (CG))	Informal caregivers of adults with an NCD	Care service telehealth
**HEALTHCARE PROVIDERS (HCPs)**							
Cheng et al., 2020	155	35, (24–54)	106 (68.4%)	Daily mood, feelings, gains and issues	DMI, SUF, GIS	T0: baseline, T1: 6 weeks	Mood improved in those who had significantly higher perceived gains (*p* < 0.001) and positive attitudes, and fewer issues
Giordano et al., 2020	34	31.8 (8.33), 22–59	22 (64.7%)	Stress, well-being	MTC	T0: baseline, T1: 1 week, T2: 2 weeks, T3: 3 weeks, T4: 4 weeks, T5: 5 weeks	T0–T1: tiredness, sadness, fear, and worry intensity decreased significantly with the BP (*p* < 0.05) T0–T1: tiredness, sadness, and worry intensity decreased significantly with the EP (*p* < 0.05) T0–T5: perceived sadness (*p* < 0.03), fear (*p* < 0.001), and worry (*p* < 0.006) decreased significantly with the BP T0–T5: perceived tiredness (*p* < 0.0001), sadness (*p* < 0.002), fear (*p* < 0.003), and worry intensity (*p* < 0.04) decreased significantly with the EP
**INFORMAL CAREGIVERS**							
De Luca et al., 2021	25	55 (12); 24–74	8 (32%)	Anxiety, perceived care burden	SAS, ZBI-22	T0: baseline, T1: 12 weeks	Anxiety and perceived care burden reduced significantly (*p* < 0.0001)
Guo et al., 2020	508 (IG = 254; CG = 254)	IG = 47.0 (4.3); CG = 46.6 (4.2)	IG = 214 (84.3%) CG = 213 (83.9%)	Depression, anxiety	PHQ-9, GAD-7	T0: baseline, T1: 4 weeks	T0–T1: no significant effect was observed on depression and anxiety Caregivers of older patients (*p* = 0.037) and not living with patients (*p* = 0.006) had lower PHQ-9 scores, caregivers of patients with a shorter duration of illness (*p* = 0.041) had lower GAD-7 scores
Lai et al., 2020	60 (IG = 30; CG = 30)	IG = 72.43 (0.80), 66–82; CG = 71.83 (0.80), 66–82	IG = 17 (56.6%) CG = 18 (60%)	QoL, perceived care burden, self-efficacy	SF-36V2, ZBI; RCSES	T0: baseline, T1: 4 weeks	T0–T1: SF-36v2, ZBI, RCSES scores increased significantly in the IG (*p* < 0.001)

Only significant *p* values were reported.

## Data Availability

Not applicable.

## Supplementary Materials

The following is available online at https://www.mdpi.com/article/10.3390/ijerph18136939/s1, Table S1: Quality Assessment Tool for Quantitative Studies Dictionary.
