# Supplementary material for: Psychological Support Interventions for Healthcare Providers and Informal Caregivers during the COVID-19 Pandemic: A Systematic Review of the Literature
Source: Int J Environ Res Public Health. 2021 Jun 28;18(13):6939. doi: 10.3390/ijerph18136939 (PMC8297206; doi:10.3390/ijerph18136939)
Supplement: Supplementary file 1 [file ijerph-18-06939-s001.zip › ijerph-1231314-supplementary.pdf]

S1: Quality Assessment Tool for Quantitative Studies Dictionary

#	ITEM	RECORDS				
		HEALTH CARE PROVIDERS (HCPs)		INFORMAL CAREGIVERS (ICs)		
		**Cheng et al. (2020)**	**Giordano et al. (2020)**	**De Luca et al. (2021)**	**Guo et al. (2020)**	**Lai et al. (2020)**
	**A - SELECTION BIAS**					
Q1	Are the individuals selected to participate in the study likely to be representative of the target population?	2 - Somewhat likely	2 - Somewhat likely	2 - Somewhat likely	2 - Somewhat likely	2 - Somewhat likely
Q2	What percentage of selected individuals agreed to participate?	5 - Can't tell	1 - 80-100%	1 - 80-100%	1 - 80-100%	1 - 80-100%
	Rate of the section	2	2	2	2	2
	**B - STUDY DESIGN**					
Q1	Indicate the study design	5 - Cohort (1 group, pre+post)	5 - Cohort (1 group, pre+post)	5 - Cohort (1 group, pre+post)	3 - Cohort analytic (2 groups, pre+post)	3 - Cohort analytic (2 groups, pre+post)
Q2	Was the study described as randomized? If NO, go to Component C.	No	No	No	No	No
Q3	If Yes, was the method of randomization described? (See dictionary)	NR	NR	NR	NR	NR
Q4	If Yes, was the method appropriate? (See dictionary)	NR	NR	NR	NR	NR
	Rate of the section	2	2	2	2	2
	**C - CONFOUNDERS**					

Q1	Were there important differences between groups prior to the intervention?	2 - No	2 - No	2 - No	1 - Yes	2 - No
Q2	If yes, indicate the percentage of relevant confounders that were controlled (either in the design (e.g. stratification, matching) or analysis)?	NR	NR	NR	1 - 80-100% (most)	NR
	Rate of the section	1	1	1	1	1
	**D - BLINDING**					
Q1	Was (were) the outcome assessor(s) aware of the intervention or exposure status of participants?	3 - Can't tell	3 - Can't tell	3 - Can't tell	3 - Can't tell	3 - Can't tell
Q2	Were the study participants aware of the research question?	3 - Can't tell	3 - Can't tell	3 - Can't tell	3 - Can't tell	3 - Can't tell
	Rate of the section	2	2	2	2	2
	**E - DATA COLLECTION METHODS**					
Q1	Were data collection tools shown to be valid?	3 - Can't tell	3 - Can't tell	1 - Yes	1 - Yes	1 - Yes
Q2	Were data collection tools shown to be reliable?	3 - Can't tell	3 - Can't tell	1 - Yes	1 - Yes	1 - Yes
	Rate of the section	3	3	1	1	1
	**F - WHITDRAWLS AND DROPOUTS**					
Q1	Were withdrawals and drop-outs reported in terms of numbers and/or reasons per group?	2 - No	2 - No	1 - Yes	1 - Yes	3 - Can't tell

Q2	Indicate the percentage of participants completing the study. (If the percentage differs by groups, record the lowest).	1 - 80-100%	1 - 80-100%	1 - 80-100%	3 - Less than 60%	1 - 80-100%
	Rate of the section	1	1	1	3	1
**G - INTERVENTION INTEGRITY**						
Q1	What percentage of participants received the allocated intervention or exposure of interest?	1 - 80-100%	1 - 80-100%	1 - 80-100%	1 - 80-100%	3 - Less than 60%
Q2	Was the consistency of the intervention measured?	2 - No	2 - No	2 - No	2 - No	2 - No
Q3	Is it likely influence that subjects received an unintended intervention (contamination or co-intervention) that may the influence results?	4 - Yes	4 - Yes	6 - Can't tell	5 - No	5 - No
**H - ANALYSIS**						
Q1	Indicate the unit of allocation (circle one)	community	community	community	community	community
Q2	Indicate the unit of analysis (circle one)	community	community	community	community	community
Q3	Are the statistical methods appropriate for the study design?	2 - No	1 - Yes	1 - Yes	1 - Yes	1 - Yes
Q4	Is the analysis performed by intervention allocation status (i.e. intention to treat) rather than the actual intervention received?	2 - No	3 - Can't tell	3 - Can't tell	1 - Yes	2 - No
**FINAL DECISION:**		MODERATE	MODERATE	STRONG	MODERATE	STRONG

Legend: 1=strong; 2=moderate; 3=weak; NR=Not Reported.
